# Needs of Young African Neurosurgeons and Residents: A Cross-Sectional Study

**DOI:** 10.3389/fsurg.2021.647279

**Published:** 2021-05-28

**Authors:** Ulrick S. Kanmounye, Faith C. Robertson, Nqobile S. Thango, Alvin Nah Doe, Nourou Dine Adeniran Bankole, Pape Aicha Ginette, Solomon Ondoma, James A. Balogun, Isabella Opoku, Luxwell Jokonya, Thioub Mbaye, Zarina A. Shabhay, Ahmed M. Ashour, Ana Cristina Veiga Silva, Beverly Cheserem, Claire Karekezi, Fahd Derkaoui Hassani, Nesrine Mentri, Tsegazeab Laeke, Abenezer Tirsit Aklilu, Samuila Sanoussi, Aaron Musara, Jeff Ntalaja, Peter Ssenyonga, Souad Bakhti, Najia El Abbadi, Muhammad Raji Mahmud, Nasser M. F. El-Ghandour, Amro Al-Habib, Angelos G. Kolias, Franco Servadei, Graham Fieggen, Mahmood Qureshi, Ignatius Esene, CAANS Young Neurosurgeons Committee and WFNS Young Neurosurgeons Committee

**Affiliations:** ^1^Research Department, Association of Future Africa Neurosurgeons, Yaoundé, Cameroon; ^2^Department of Neurosurgery, Massachusetts General Hospital, Boston, MA, United States; ^3^Division of Neurosurgery, Department of Surgery, University of Cape Town, Cape Town, South Africa; ^4^Neurosurgery Sub-Unit, Department of Surgery, John F. Kennedy Medical Center, Monrovia, Liberia; ^5^Neurosurgery Department, Centre Hospitalier Universitaire Ibn Sina Rabat- Mohamed V University of Rabat, Rabat, Morocco; ^6^Division of Neurosurgery, Felix Houphouet Boigny University of Abidjan, Abidjan, Côte d'Ivoire; ^7^Mercy One Neurosurgery, Mercy One Hospital of North Iowa, Mason, IA, United States; ^8^Division of Neurological Surgery, Department of Surgery, College of Medicine, University of Ibadan, Ibadan, Nigeria; ^9^Department of Neurosurgery, China International Neuroscience Institute (China-INI), Beijing, China; ^10^Division of Neurosurgery, College of Health Sciences, University of Zimbabwe, Harare, Zimbabwe; ^11^Department of Neurosurgery, Centre Hospitalier Universitaire Fann, Dakar, Senegal; ^12^Division of Neurosurgery, Department of Surgery, Muhimbili Orthopedic Institute, Dar es Salaam, Tanzania; ^13^Department of Neurosurgery, Ain Shams University, Cairo, Egypt; ^14^Neurosurgery Department, Restauração Hospital, Recife, Brazil; ^15^Department of Neurosurgery, Aga Khan University Hospital, Nairobi, Kenya; ^16^Neurosurgery Unit, Department of Surgery, Rwanda Military Hospital, Kigali, Rwanda; ^17^Department of Neurosurgery, Cheikh Zaid International Hospital, Abulcasis International University of Health Sciences, Rabat, Morocco; ^18^Department of Neurosurgery, Bejaia University Hospital, Béjaïa, Algeria; ^19^Neurosurgery Unit, Department of Surgery, College of Health Sciences, Addis Ababa University, Addis Ababa, Ethiopia; ^20^Department of Neurosurgery, Niamey National Hospital, Niamey, Niger; ^21^Department of Neurosurgery, University of Lubumbashi, Lubumbashi, Democratic Republic of Congo; ^22^Department of Neurosurgery, CURE Children's Hospital of Uganda, Mbale, Uganda; ^23^Pediatric Neurosurgery Division, Department of Neurosurgery, Academic Hospital Mustapha Pacha, Algiers, Algeria; ^24^Neurosurgery Unit, Department of Surgery, Ahmadu Bello University, Zaria, Nigeria; ^25^Department of Neurosurgery, Faculty of Medicine, Cairo University, Cairo, Egypt; ^26^Division of Neurosurgery, Department of Surgery, College of Medicine, King Saud University, Riyadh, Saudi Arabia; ^27^NIHR Global Health Research Group on Neurotrauma, University of Cambridge, Cambridge, United Kingdom; ^28^Department of Neurosurgery, Humanitas University and Research Hospital, Milan, Italy; ^29^Division of Neurosurgery, Neurosciences Institute, Faculty of Health Sciences, University of Cape Town, Cape Town, South Africa; ^30^Neurosurgery Division, Faculty of Health Sciences, University of Bamenda, Bambili, Cameroon

**Keywords:** Africa, education, global neurosurgery, neurosurgery, research

## Abstract

**Introduction:** Africa has many untreated neurosurgical cases due to limited access to safe, affordable, and timely care. In this study, we surveyed young African neurosurgeons and trainees to identify challenges to training and practice.

**Methods:** African trainees and residents were surveyed online by the Young Neurosurgeons Forum from April 25th to November 30th, 2018. The survey link was distributed via social media platforms and through professional society mailing lists. Univariate and bivariate data analyses were run and a *P*-value < 0.05 was considered to be statistically significant.

**Results:** 112 respondents from 20 countries participated in this study. 98 (87.5%) were male, 63 (56.3%) were from sub-Saharan Africa, and 52 (46.4%) were residents. 39 (34.8%) had regular journal club sessions at their hospital, 100 (89.3%) did not have access to cadaver dissection labs, and 62 (55.4%) had never attended a WFNS-endorsed conference. 67.0% of respondents reported limited research opportunities and 58.9% reported limited education opportunities. Lack of mentorship (*P* = 0.023, Phi = 0.26), lack of access to journals (*P* = 0.002, Phi = 0.332), and limited access to conferences (*P* = 0.019, Phi = 0.369) were associated with the country income category.

**Conclusion:** This survey identified barriers to education, research, and practice among African trainees and young neurosurgeons. The findings of this study should inform future initiatives aimed at reducing the barriers faced by this group.

## Introduction

Although low- and middle-income countries (LMICs) have the greatest burden of neurosurgical diseases, their access to resources is limited (1). Neurosurgeons in these regions, especially in Africa, face unique challenges (2). African countries have some of the highest cases per neurosurgeon, but few patients live within 2-h of a neurosurgical center (3–5). Moreover, most African patients, do not have access to comprehensive health insurance (6). The resulting out-of-pocket expenditures expose them to catastrophic and impoverishing expenditures and limit their access to surgical care (7).

Professional societies are supporting initiatives aimed at improving access to neurosurgical care in Africa. For example, The World Federation of Neurosurgical Societies (WFNS) sponsors the training of African neurosurgeons in accredited reference training centers (8) and the Continental Association of African Neurosurgical Societies (CAANS) recently created an *ad-hoc* committee to assist residents and young neurosurgeons. The Young African Neurosurgeons Committee has been tasked with facilitating education and research among future and young African neurosurgeons.

To understand the barriers and facilitators of research and education in African neurosurgery, the Young African Neurosurgeons Committee and the Young Neurosurgeons Forum of the WFNS (YNF-WFNS) surveyed residents, fellows, and consultants who are within 10 years of completing residency (9, 10). In this paper, we aimed to assess the needs and challenges faced by young African neurosurgeons and residents in their daily clinical and research activities.

## Methods

This was a cross-sectional study consisting of a self-administered survey composed of 28 multiple-choice and two free-text questions (Appendix 1) on the respondents' demographics, the type of neurosurgical center they worked in, access to infrastructures, facilitators, and barriers of research and education in daily practice, and suggested solutions (9, 10). The e-survey was developed and piloted by members of the YNF-WFNS. It respected the Checklist for Reporting Results of Internet E-Surveys (CHERRIES) guidelines and its face validity was established by WFNS officials.

A cover letter was annexed to the survey and data were collected from April 25th to November 30th, 2018. The survey link (Qualtrics, USA) was distributed to the electronic mailing lists of the YNF-WFNS and Young CAANS and to personal contacts via email and instant messages on social media platforms (Twitter, Facebook, and WhatsApp).

Respondents were a convenience sample of neurosurgeons and residents, and their responses to the survey were limited to one. The participation and dropout rates were not computed. Chi-Squared test, Pearson's Phi coefficient measure, and non-parametric multivariable tests were used to analyze the data, and the threshold of significance was set at 0.05. Data analysis was done using SPSS v. 26 (IBM, USA).

## Results

### Demographics and Resources

**Figure 1:**
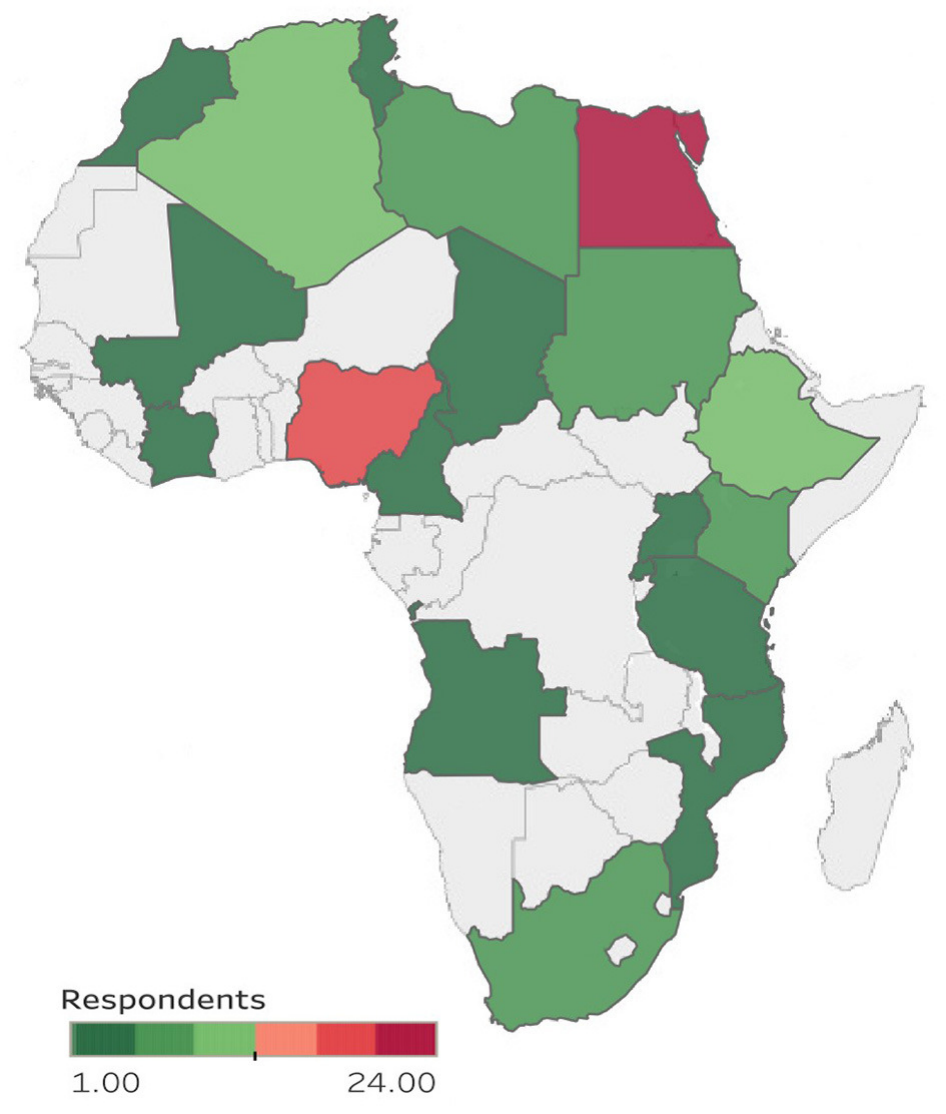
Countries represented in the e-survey and the number of respondents per country.

Out of the 280 Young CAANS members, 112 neurosurgeons and residents from 20 African countries responded to the survey i.e., a response rate of 40% (Figure 1). There were no partially completed survey responses.

Sixty-six (58.9%) were from lower-middle-income countries, and 63 (56.3%) were from Sub-Saharan Africa. Ninety-eight (87.5%) respondents were male, 79 (70.5%) were aged between 30 and 40 years, and 52 (46.4%) were neurosurgery residents. Although 76 respondents (67.9%) worked in a university teaching hospital, only 33 (29.5%) declared being paid to do clinical work and research. Most respondents (71.4%) worked in cities of more than 1.5 million inhabitants (Table 1).

**Table 1:** Descriptive characteristics of the survey respondents and their activities.

**Characteristic**	**Number of respondents [*n* (%)]**
	***N* = 112**
**Sex**	
Female	14 (12.5)
Male	98 (87.5)
**Age (Years)**	
< 30	19 (17.0)
30-35	51 (45.5)
36–40	28 (25.0)
≥41	14 (12.5)
**Region**	
North Africa	49 (43.8)
Sub-Saharan Africa	63 (56.3)
**World Bank income category**	
Low-income	20 (17.9)
Lower-middle-income	66 (58.9)
Upper-middle-income	26 (23.2)
**Profession**	
Resident (< 5 years after graduating from medical school)	20 (17.9)
Resident (5 years or more after graduating from medical school)	32 (28.6)
Fellow	27 (24.1)
Consultant < 5 years after finishing residency	12 (10.7)
Consultant 5 years or more after finishing residency	17 (15.2)
Other	4 (3.6)
**Population of the city respondents work in**	
< 50,000	2 (1.8)
200,000–500,000	8 (7.1)
500,000–1,500,000	22 (19.6)
>1,500,000	80 (71.4)
**Paid activities**	
Clinical	79 (70.5)
Clinical and research	33 (29.5)

The majority of the respondents' hospitals had a capacity of 500 or fewer beds (55.4%) and 80 respondents (71.4%) reported they had dedicated neurosurgical wards. Most respondents reported having operating microscopes (68.8%) and intensive care units equipped with ventilators (91.1%). One hundred and nine (97.3%) had access to CT scans, and 88 individuals (78.6%) had access to MRIs. Only 31 (27.7%) respondents had access to catheter angiography. A summary of these data can be found in Table 2.

**Table 2:** Availability of material and human resources.

**Resource**	**Number (Percentage)**
**Hospital**
Mixed activity (public and private)	15 (13.4)
Non-teaching public hospital	20 (17.9)
Private hospital	1 (0.9)
University teaching hospital	76 (67.9)
**Bed capacity**
≤ 500	62 (52.4)
500–1,000	32 (28.6)
>1,000	18 (16.1)
Units that share their ward with other specialties	80 (71.4)
**Number of ward beds dedicated to neurosurgery**
< 25	31 (27.7)
25–50	47 (42.0)
50–75	19 (17.0)
75–100	8 (7.1)
>100	7 (6.3)
ICUs without mechanical ventilators	10 (8.9)
**Equipment**
Catheter angiography	31 (27.7)
CT Scan	109 (97.3)
High-speed drill	65 (58.0)
Image guidance	27 (24.1)
MRI	88 (78.6)
Operating microscope	77 (68.8)
Rehabilitation specialists	57 (50.9)

**Figure 2:**
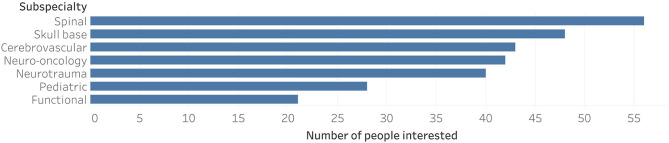
Subspecialty interests.

The most popular subspecialties where spine (50%; 95% CI: 40.4–59.6%), skull base (42.9%; 95% CI: 33.5–52.6%), and cerebrovascular surgery (38.4% 95% CI: 29.4–48.1%) (Figure 2). Spine surgery was more popular among residents than fellows or consultants (61.5 vs. 47.6 vs. 35.9%, respectively, *P* = 0.05). In contrast, cerebrovascular surgery was more popular among fellows than residents and consultants (52.4 vs. 46.2 vs. 20.5%, respectively, *P* = 0.02). The other subspecialty popularity differences did not show statistical significance.

### Challenges to Education and Research

Only 39 (34.8%) respondents had journal clubs at their institutions, and 100 (89.3%) did not have access to hands-on cadaver dissection. Sixty two participants (55.4%) had never attended a WFNS conference or WFNS sponsored meeting,

### Perceived Barriers

Almost every respondent (94.6%) felt that the neurosurgical needs of their local population were not adequately covered. The limited number of ICU beds (72.3%), lack of access to microsurgical equipment (59.8%) and inadequate/no insurance coverage (56.3%) were identified as major barriers to a suitable neurosurgical coverage of the patient population. Two out of three respondents reported limited opportunity to do research (67.0%), 66 reported limited access to organized teaching and training sessions (58.9%) (Table 3). Most participants reported hands-on courses as their preferred method of training (91.1%), 80 respondents preferred personal attendance (71.4%), and less than half (44.6%) chose web-based lectures.

**Table 3:** Perceived barriers to day-to-day practice.

**Barrier**	**Frequency (Percentage)**
Inadequate or no insurance coverage	63 (56.3)
Limited number of trained neurosurgeons	57 (50.9)
Limited number of neurosurgical beds	52 (46.4)
Limited number of ICU beds	81 (72.3)
Lack of access to equipment necessary for microsurgery	67 (59.8)
Lack of regular/consistent access to CT	19 (17.0)
Lack of regular access to MRI	45 (40.2)
Lack of organized primary care	46 (41.1)
Lack of organized pre-hospital/emergency hospital care	60 (53.6)
Lack of organized rehabilitation care	61 (54.5)
Lack of access to organized teaching/training sessions	66 (58.9)
Limited number of opportunities for hands-on operating	58 (51.8)
Long hours of work	48 (42.9)
Poor work/life balance	63 (56.3)
Bullying and harassment issues	18 (16.1)
Lack of regular access to the advice of experienced/senior colleagues	38 (33.9)
Lack of a mentor	34 (30.4)
Lack of access to neurosurgical journals	50 (44.6)
Lack of access to neurosurgical textbooks	25 (22.3)
Limited opportunities to do research	75 (67.0)

The following hurdles in daily neurosurgical practice and the personal needs of our participants were found to be associated with the World Bank Income Class classification: inadequate or lack of insurance coverage (*P* < 0.001, Phi = 0.498), limited number of trained neurosurgeons (*P* < 0.001, Phi = 0.375), limited number of neurosurgical beds (*P* = 0.003, Phi = 3.24), lack of access to equipment (*P* = 0.004, Phi = 0.314), lack of organized prehospital care (*P* = 0.005, Phi = 0.309), lack of regular access to the advice of senior colleagues (*P* = 0.002, Phi = 0.335), lack of a mentor (*P* = 0.023, Phi = 0.26), lack of access to journals (*P* = 0.002, Phi = 0.332) and limited attendance at a neurosurgical conference (*P* = 0.019, Phi = 0.369).

More West African respondents reported bullying and harassment than their counterparts from South, East, North, and Central Africa (43.5 vs. 14.3 vs. 13.3 vs. 6.1 vs. 0%, respectively, *P* = 0.002). However, more Central African respondents experienced difficulties accessing journals than respondents from West, East, North, and South Africa (100 vs. 56.5 vs. 53.3 vs. 36.7 vs. 0%, respectively, *P* = 0.01). Also, more fellows reported limited research opportunities than consultants and residents (85.7 vs. 76.9 vs. 51.9%, respectively, *P* = 0.01).

## Discussion

This is the first study to examine the needs of African neurosurgery specialists and trainees. African residents and consultant neurosurgeons felt the neurosurgical needs of their patients were not met entirely. Respondents faced numerous barriers to neurosurgical practice, education, and research.

### Practice and Education

African neurosurgery residents and practitioners had relatively high access to basic neuroimaging. 97.3% had access to a CT scan, and 78.6% had access to an MRI. In a survey of African neurosurgical residents regarding the adequacy of their training, Sader et al. found similar numbers - 95% for CT-scans and 80% for MRIs (11). In contrast, access to neurorehabilitation and operative equipment was limited. Only 58.0% of respondents had access to high-speed drills, 50.9% had access to neurorehabilitation services, and 24.1% had access to neuronavigation. It is concerning that only 27.7% of respondents had access to catheter angiography, given the enormous and rapidly increasing burden of cerebrovascular diseases in Africa (12–14). The scarcity of neurorehabilitation services and operative equipment concerns because 57.2% of participants were either residents or consultant neurosurgeons with < 5 years of experience, and 67.9% worked at university teaching hospitals. With 91.0% of respondents working in cities with at least 500,000 inhabitants, African residents and early career neurosurgeons are therefore working in under-resourced high-volume centers. This limits their training and professional development. Neurorehabilitation and operative equipment have previously been identified as limiting factors to the development of neurosurgical practice in low-resource settings (11, 15–17). It is therefore crucial that strategies aimed at increasing the neurosurgical workforce in Africa are accompanied by investments in equipment and neurorehabilitation.

The benefits of neurosurgical dissection are undeniable. Participation in dissection labs increases the understanding of neuroanatomical relationships and improves operative skills (18, 19). For these reasons, hands-on dissections have become an integral part of postgraduate neurosurgical training (20).

Residency programs and professional societies in high-income countries offer dissection labs to their residents and young neurosurgeons but 89.3% of African residents and neurosurgeons did not have dissection labs at their home institutions. The cost of dissection labs can be prohibitive for LMIC neurosurgical centers and, in effect, constitutes a barrier to the training of neurosurgeons. Moreover, 55.4% of respondents had never been to an international neurosurgical event and were, therefore, less likely to have participated in a cadaver dissection abroad.

African neurosurgeons are aware of these difficulties and are working locally and internationally to improve service delivery and practice. Locally, South African training centers like the University of Cape Town offer sponsored pediatric neurosurgery fellowships to African neurosurgeons (21). Internationally, African training centers partner with non-governmental and academic organizations from high-income countries. For example, the Foundation for International Education in Neurological Surgery (FIENS) provides funded fellowships in American high volume academic centers to young African neurosurgeons (22). Also, East African training centers have collaborated with the Neurosurgery Education and Development (NED) foundation to provided endoscopic treatment and training using a mobile endoscopy unit (23). In Tanzania and Uganda, Weill Cornell and Duke University are running global neurosurgery fellowships in partnership with local training programs (24). Furthermore, in West Africa, Nigerian and Swedish centers have expanded the local neurosurgical capacity following the successful implementation of a twinning program (25).

While these collaborations provide valuable experience, few provide exposure to cadaver dissections. A solution around this is the use of low-cost, high-fidelity solutions for dissection. These include the use of veterinary cadavers for spinal dissection, use of gelatin and silicone to simulate cerebrovascular surgery dissection, and the use of phone cameras as operative microscopes (26–28). These cheaper and innovative solutions can bridge the training gap in African neurosurgery programs.

The ongoing COVID pandemic has changed the landscape of neurosurgical education on the African continent, limiting physical interactions and in-person conferences. Educators have developed online solutions to meet the educational needs of African trainees and residents. These online events have the advantage of being less expensive because they eliminate travel and visa costs but they present a unique set of challenges. Online sessions offer less face-to-face and hands-on time in comparison with physical conferences and symposia. As such, they cannot be a substitute to in-person and hands-on experiences but they are and should be complements.

### Research

African residents and young neurosurgeons equally face significant challenges in research. While it is true that the contribution of Africa to global neurosurgical research has increased over the past two decades, it still has a long way to go (29). One of the barriers to African neurosurgical research is protected time. Protected time is indispensable for the development of neurosurgeon-scientists (30). Most American residency programs encourage their residents to pursue research activities. Most American residents have a year or more of protected research time (31). Similarly, 45.0% of residents in Latin America are enrolled in a program with protected research time (32). These figures contrast starkly with 29.5% of residents in Africa who have protected research time.

In addition, 65.2% of participants did not have journal clubs at their institutions. In contrast, 85% of American residents have journal clubs at their training programs (33). Journal clubs expose participants to recent literature, enhance critical appraisal skills, and facilitate the practice of evidence-based neurosurgery (34). Given the high volume of neurosurgical literature and the heavy workload of neurosurgeons and residents, journal clubs are an opportunity for participants to keep up with the literature. Journal clubs should be organized on a monthly basis, and articles should be selected based on their impact on everyday practice (35).

### Proposed Solutions

Seminar courses and workshops are crucial to the training of young African neurosurgeons (2). The Young African Neurosurgeons Committee has been organizing research courses at regional meetings to build research capacity among residents and young neurosurgeons (36, 37). Digital education modules, operative videos, and telesimulation are effective in tackling the current research and education barriers in low resource settings (38). The YNF-WFNS organizes live webinars for all young neurosurgeons (39). Moreover, as a result of the COVID-19 pandemic, most academic institutions have switched to online education solutions and have opened their webinars to non-members (40). These webinars facilitate education, peer mentorship, and collaboration among participants. Telesimulation is a good complement to hands-on courses. It reduces geographical barriers but cannot substitute hands-on lab courses.

It is unlikely that every African neurosurgical center will have all the necessary resources in the near future. In the meantime, these centers must leverage inter-African partnerships and collaborations with non-African institutions to tackle the challenges to research and education that we have identified. The partnerships between LMIC and high-income institutions must be based on equity and have frequent monitoring and evaluation (41, 42). Additionally, it is equally important to coordinate efforts so as to avoid redundancy.

### Limitations

Young neurosurgeons and trainees without reliable internet, electronic devices, and email are less likely to be captured. Moreover, our use of non-randomized sampling methods reduces the external validity of our survey findings. Notwithstanding, our survey is the first to assess the needs and barriers faced by Young African researchers and with representatives of 20 African countries, we believe our findings are close to the truth. Also, our survey predated the COVID-19 pandemic and as such did not capture the pandemic-related needs of African residents and young neurosurgeons. This is a limitation considering that the pandemic led to cancellation of rotations, fellowships, and physical conferences. By presenting a pre-pandemic overview of the needs and barriers faced by African trainees and neurosurgeons, our study provides data to better appreciate the impact of the pandemic. We believe this data will be informative for the WFNS, CAANS, and their partners as they determine solutions to mitigate the impact of the pandemic on African neurosurgery.

## Conclusion

There has been evolution in neurosurgical practice in Africa over the past two decades however neurosurgeons and residents face a myriad of difficulties in their day-to-day practice and education. Current efforts by the WFNS, CAANS, Young CAANS and YNF should be encouraged and the efforts of these organizations should prioritize actions that tackle the problems identified by young African neurosurgeons, namely the dearth of access to research, skills labs, literature and mentorship.

## Data Availability Statement

The raw data supporting the conclusions of this article will be made available by the authors, without undue reservation.

## Ethics Statement

The studies involving human participants were reviewed and approved by World Federation of Neurosurgical Societies. Written informed consent for participation was not required for this study in accordance with the national legislation and the institutional requirements.

## Author Contributions

UK and FR: methodology, investigation, data curation, and writing - original draft. AD, NB, NT, PG, SO, JB, IO, LJ, TM, ZS, BC, CK, SS, AM, JN, PS, SB, NE, MM, NE-G, FS, GF, and MQ: conceptualization and writing - review & editing. AMA, AS, FH, NM, and TL: conceptualization, investigation, and writing - review & editing. AA-H, AK, and IE: conceptualization, methodology, investigation, and writing - review & editing. IE: conceptualization, methodology, investigation, data curation, and writing - review & editing. All authors contributed to the article and approved the submitted version.

## Conflict of Interest

The authors declare that the research was conducted in the absence of any commercial or financial relationships that could be construed as a potential conflict of interest.
