# Supplementary material for: Needs of Young African Neurosurgeons and Residents: A Cross-Sectional Study
Source: Front Surg. 2021 May 28;8:647279. doi: 10.3389/fsurg.2021.647279 (PMC8193351; doi:10.3389/fsurg.2021.647279)
Supplement: Supplementary file 1 [file Data_Sheet_1.DOCX]

Supplementary Material

Survey Questions

WFNS YN Survey

Start of Block: Default Question Block

Start of Block: Block 4

Q1 Name:  (Optional)

________________________________________________________________

| 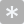 |
| --- |

Q2 Email address if you want to be notified about the committee’s activities:  (Optional)

________________________________________________________________

Q3 Age

- < 30 (1)
- 30-35 (2)
- 36-40 (3)
- 41 or more (4)

Q4 Gender

- Male (1)
- Female (2)

| 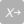 |
| --- |

Q5 In which country do you currently reside?

▼ Afghanistan (1) ... Zimbabwe (1357)

Q6 What is the size of the town/city in which you work?  (Optional)

- >1.5 million (1)
- 500 000 - 1.5 million (2)
- 200 000 - 500 000 (3)
- 50 000 - 200 000 (4)
- <50 000 (5)

Q7 Level of practice:

- Resident (< 5 years after graduating from medical school) (1)
- Resident (5 years or more after graduating from medical school) (2)
- Fellow (defined as additional training near the end or after the end of residency) (3)
- Consultant < 5 years after finishing residency (4)
- Consultant 5 years or more after finishing residency (5)
- Other (6) ________________________________________________

Q8 What is your main subspecialty (for residents, which subspecialty are you interested in)?

- Cerebrovascular Surgery (1)
- Functional Neurosurgery (2)
- Neuro-endoscopy (3)
- Neuro-oncology (4)
- Neurotrauma (5)
- Paediatric Neurosurgery (6)
- Skull Base Surgery (7)
- Spinal Surgery (8)
- Other (9) ________________________________________________

Q9 Is your appointment (job) purely clinical or are you also paid by your employer to do research?

- Clinical (1)
- Clinical and I am paid to do research (2)
- Research only (3)

End of Block: Block 4

Start of Block: Block 1

Q10 How would you describe your main place of work?

- University / Teaching Hospital (1)
- Other Public Hospital (2)
- Private Hospital (3)
- Mixed Public and Private Hospital (4)

Q11 Number of hospital beds:

- 500 or less beds (1)
- 500 to 1000 beds (2)
- More than 1000 beds (3)

Q12 Are there dedicated neurosurgical wards in your hospital?  (This does not include high dependency or intensive care unit beds)

- Yes (1)
- No (i.e. we share wards with other specialties) (2)

Q13 Number of neurosurgical beds (Including beds in wards, high dependency unit, and intensive care unit, if applicable):

- < 25 (1)
- 25-50 (2)
- 50-75 (3)
- 75-100 (4)
- > 100 (5)

Q14 Which of these imaging modalities do you have access to?

- CT scan (1)
- MRI (2)
- Catheter Angiography (3)

Q15 Do you have an operating microscope?

- Yes (1)
- No (2)

Q16 Do you have an image guidance system (navigation)?

- Yes (1)
- No (2)

Q17 Do you have a high-speed drill?

- Yes (1)
- No (2)

Q18 Do you have access to Intensive Care Unit (ICU) Beds?

- Yes (1)
- No (2)

Q19 Do you have access to mechanical ventilators in the ICU?

- Yes (1)
- No (2)

Q20 Do you have access to specialists in rehabilitation?

- Yes (1)
- No (2)

End of Block: Block 1

Start of Block: Education/Training

Q21 Do you have time dedicated for neurosurgical education (didactic teaching, topic presentations etc) during the week?

- Yes (1)
- No (2)

Q22 Does your department hold a journal club?

- Yes (1)
- No (2)

Q23 Do you have access to regular hands-on cadaver training courses in your department?

- Yes (1)
- No (2)

Q24 In your view, what are the biggest hurdles that are not letting your neurosurgical service (i.e. the hospital you are working at) provide neurosurgical care that can satisfy the needs of your local population in its entirety (you can select > 1)?

- N/A - the neurosurgical care needs of my local population are perfectly covered (1)
- Inadequate or no insurance coverage for significant number of people (2)
- The limited number of trained neurosurgeons (3)
- The limited number of neurosurgical beds (4)
- The limited number of ICU beds (5)
- Lack of access to equipment necessary for microsurgery (e.g. microscope, drill, bipolar) (6)
- Lack of regular / consistent access to CT (7)
- Lack of regular access to MRI (8)
- Lack of organised primary care (9)
- Lack of organised pre-hospital / emergency hospital care (10)
- Lack of organised rehabilitation care (11)
- Other (12) ________________________________________________

Q25 In your view, what are the biggest hurdles in your own personal day-to-day practice (you can select > 1)?

- N/A-there are no hurdles (12)
- Lack of access to organised teaching / training sessions (1)
- Limited number of opportunities for hands-on operating (2)
- Long hours of work (3)
- Poor work / life balance (4)
- Bullying and harassment issues (5)
- Lack of regular access to the advice of experienced / senior colleagues (6)
- Lack of a mentor (7)
- Lack of access to neurosurgical journals (8)
- Lack of access to neurosurgical textbooks (9)
- Limited opportunities to do research (10)
- Other (11) ________________________________________________

Q26 Are you a member of a national neurosurgical society?

- Yes (1)
- No (2)

Display This Question:

If Are you a member of a national neurosurgical society? = Yes

Q27 If yes, which one?

________________________________________________________________

Q28 Have you attended a WFNS conference or a WFNS supported meeting before?

- Never (1)
- Once (2)
- Twice (3)
- More than 2 times (4)

Q29 Please list three areas you would like the WFNS help you in your personal goals and the goals of your neurosurgical service (e.g. education, research etc)?  (Optional)

- Area 1 (1) ________________________________________________
- Area 2 (2) ________________________________________________
- Area 3 (3) ________________________________________________

Q30 Assuming that you are interested in attending education / training provided by the WFNS, which of these formats suit you?

- Web-based lectures (1)
- Personal attendance in a course or a conference (2)
- Hands-on course (3)

End of Block: Education/Training

Start of Block: Block 3
